# Aberrantly expressed PLOD1 promotes cancer aggressiveness in bladder cancer: a potential prognostic marker and therapeutic target

**DOI:** 10.1002/1878-0261.12532

**Published:** 2019-06-27

**Authors:** Yasutaka Yamada, Mayuko Kato, Takayuki Arai, Hiroki Sanada, Akifumi Uchida, Shunsuke Misono, Shinichi Sakamoto, Akira Komiya, Tomohiko Ichikawa, Naohiko Seki

**Affiliations:** ^1^ Department of Functional Genomics Chiba University Graduate School of Medicine Japan; ^2^ Department of Urology Chiba University Graduate School of Medicine Japan; ^3^ Department of Pulmonary Medicine Graduate School of Medical and Dental Sciences Kagoshima University Japan

**Keywords:** bladder cancer, inhibitor, microRNA, *miR‐140‐5p*, passenger strand, PLOD1

## Abstract

Bladder cancer (BC) is the ninth most malignant tumor worldwide. Some BC patients will develop muscle‐invasive BC (MIBC), which has a 5‐year survival rate of approximately 60% due to metastasis. As such, there is an urgent need for novel therapeutic and diagnostic targets for MIBC. Analysis of novel antitumor microRNA (miRNA)‐mediated cancer networks is an effective strategy for exploring therapeutic targets and prognostic markers in cancers. Our previous miRNA analysis revealed that *miR‐140‐5p* acts as an antitumor miRNA in BC cells. Here, we investigated *miR‐140‐5p* regulation of BC molecular pathogenesis. Procollagen‐lysine, 2‐oxoglutarate 5‐dioxygenase 1 (*PLOD1*) was found to be directly regulated by *miR‐140‐5p*, and aberrant expression of *PLOD1* was observed in BC clinical specimens. High *PLOD1* expression was significantly associated with a poor prognosis (disease‐free survival: *P *=* *0.0204; overall survival: *P *=* *0.000174). Multivariate analysis showed PLOD1 expression to be an independent prognostic factor in BC patients (hazard ratio = 1.51, *P *=* *0.0099). Furthermore, downregulation of PLOD1 by siRNAs and a specific inhibitor significantly decreased BC cell aggressiveness. Aberrant expression of PLOD1 was closely associated with BC pathogenesis. In summary, the present study showed that PLOD1 may be a potential prognostic marker and therapeutic target for BC.

**Abbreviations**
BC: bladder cancer
GEO: Gene Expression Omnibus
miRNA: microRNA
PLOD1: procollagen‐lysine, 2‐oxoglutarate 5‐dioxygenase 1
RISC: RNA‐induced silencing complex
TCGA: The Cancer Genome Atlas

## 1 Introduction

Bladder cancer (BC) is the ninth most malignant tumor worldwide, and approximately 430 000 cases were newly diagnosed in 2012 (Antoni *et al*., 2017). BC is clinically divided into two groups: muscle‐invasive BC (MIBC) and non‐muscle‐invasive BC (NMIBC) (Lemke and Shah, 2018). Patients with the latter have a favorable prognosis (5‐year survival rate: approximately 90%) after surgical resection. However, approximately 50% of cases develop intravesical recurrence after surgical resection, and approximately 15–40% of recurrent BC cases are invasive and exhibit distant metastasis (Lemke and Shah, 2018). Although radical cystectomy and cisplatin‐based combination chemotherapy are the standard treatments for MIBC, the 5‐year survival rate of patients with MIBC is approximately 60% (Chou *et al*., 2016; Lemke and Shah, 2018). In addition, the survival of patients with distant metastasis is only 15 months due to no effective treatment options (Abufaraj *et al*., 2018). Therefore, discovery of novel therapeutic and diagnostic targets is urgently needed.

A vast number of studies have shown that a large number of noncoding RNAs encoded by the human genome are functional and play critical roles in various cellular processes, for example, cell growth, migration, invasion, and apoptosis (Bartel, 2004). microRNAs (miRNAs), a class of noncoding RNAs, are endogenous single‐stranded RNA molecules comprising 19–22 nucleotides that function as fine‐tuners of RNA expression (Bartel, 2009; Goto *et al*., 2015b; Koshizuka *et al*., 2017a; Kurozumi *et al*., 2017). A single miRNA regulates a vast number of RNA transcripts, and a bioinformatics study showed that approximately 60% of protein‐coding genes are controlled by miRNAs (Bartel, 2009). Aberrantly expressed miRNAs are closely associated with cancer pathogenesis via disruption of RNA networks within cancer cells (Beermann *et al*., 2016).

Using the knowledge that a single miRNA controls numerous genes, we sequentially identified novel cancer pathways regulated by antitumor miRNAs in several cancers (Goto *et al*., 2015a, 2017; Miyamoto *et al*., 2016). Identification of dysregulated miRNAs in cancer cells is the first step, and the latest RNA‐sequencing technology is suitable for producing miRNA signatures. Interestingly, analyses of our RNA‐sequencing‐based signatures revealed that the passenger strand of some miRNAs is up‐ or downregulated in cancer tissues (Goto *et al*., 2017; Koshizuka *et al*., 2017b). During miRNA biogenesis, the passenger strand of the miRNA duplex is degraded and does not play a role in gene regulation in cells (Mah *et al*., 2010). Our recent studies revealed that the passenger strand of certain miRNAs (e.g., *miR‐99a‐3p*,* miR‐144‐5p*,* miR‐145‐3p*,* miR‐455‐5p,* and *miR‐223‐5p*) acts as an antitumor miRNA by targeting several oncogenic genes closely involved in cancer pathogenesis (Arai *et al*., 2018a,2018b; Goto *et al*., 2017; Matsushita *et al*., 2015; Sugawara *et al*., 2018; Yamada *et al*., 2018a,2018b).

Based on the miRNA signature of BC, we focused on *miR‐140‐5p* (the passenger strand of the *miR‐140*‐duplex) to investigate the function of *miR‐140‐5p* and identify its target oncogenes as therapeutic and diagnostic targets for BC. Our data showed that procollagen‐lysine, 2‐oxoglutarate 5‐dioxygenase 1 (*PLOD1*) is directly regulated by *miR‐140‐5p* in BC cells. Aberrant expression of *PLOD1* was closely associated with BC pathogenesis. Notably, inhibition of PLOD1 by transfection of siRNA or a PLOD1 inhibitor significantly attenuated the malignant phenotype of BC cells.

## 2 Materials and methods

### 2.1 Clinical specimen collection and cell culture

We obtained 15 BC tissues and normal adjacent tissues from patients undergoing total cystectomy at Chiba University Hospital between 2014 and 2015 (Table S1). All patients provided informed written consent forms, and the study protocol was approved by the Institutional Review Board of Chiba University (number: 484). The study methodologies conformed to the standards set by the Declaration of Helsinki. We used the human BC cell lines T24 and BOY. These cell lines were cultured in RPMI 1640 Medium supplemented with 10% fetal bovine serum (HyClone, Logan, UT, USA) as described previously (Yamada *et al*., 2018d).

### 2.2 Transfection of mature miRNAs, siRNAs, and plasmid vectors

We used the following agents in this study: the precursor sequences of *hsa‐miR‐140‐5p* and *hsa‐miR‐140‐3p* (assay IDs: PM10205 and PM12503, respectively; Applied Biosystems, Foster City, CA, USA), negative control miRNA (miR‐control) (assay ID: AM 17111; Applied Biosystems), and PLOD1‐specific siRNA (si*‐PLOD1*) (Stealth Select RNAi siRNA*,* P/N: HSS108122 and HSS108123; Invitrogen, Carlsbad, CA, USA). A plasmid vector containing *PLOD1* was provided by OriGene (cat. no. SC119956; Rockville, MD, USA). Transfection of the agents into cells was performed using previously described procedures (Yamada *et al*., 2018c). miRNAs and siRNAs were incubated with 10 nm Lipofectamine RNAiMax transfection reagent (Invitrogen) diluted in Opti‐MEM (Invitrogen). Plasmid vectors were incubated with Lipofectamine 3000 reagent (Invitrogen) in Opti‐MEM for forward transfection.

### 2.3 PLOD1 inhibitor studies

We used 2,2′‐dipyridyl (07‐5990; Sigma‐Aldrich, St. Louis, MO, USA), previously reported to be a small‐molecule PLOD1 inhibitor, to inhibit PLOD1 in *in vitro* assays (Jover *et al*., 2018).

### 2.4 Quantitative reverse transcription–polymerase chain reaction (qRT‐PCR)

TaqMan probes and primers specific to *PLOD1* (P/N: Hs00609363_m1; Applied Biosystems), which are assay‐on‐demand gene expression products, were used to analyze *PLOD1* expression. *miR‐140‐5p* (P/N:001187; Applied Biosystems) and *miR‐140‐3p* (P/N:002234; Applied Biosystems) expression was analyzed by qRT‐PCR. mRNA and miRNA expression levels were normalized to those of *GUSB* (P/N: Hs99999908_m1; Applied Biosystems) and *RNU48* (assay ID: 001006; Applied Biosystems). PCR quantification was performed as described previously (Yamada *et al*., 2018d).

### 2.5 Cell proliferation, migration, and invasion assays

Cell proliferation was evaluated by the XTT assay using the Cell Proliferation Kit II (Sigma‐Aldrich). Cell migration was assessed by wound healing assays, and invasion was determined using modified Boyden chambers containing Matrigel‐coated Transwell membrane filter inserts.

### 2.6 Cell‐cycle assay

Bladder cancer cells were transiently transfected with either the transfection reagent only as the control or the 2,2′‐dipyridyl, PLOD1 inhibitor, in six‐well tissue culture plates. Cells were harvested by trypsinization 72 h after transfection. For cell‐cycle analysis, cells were stained with propidium iodide using the Cycletest Plus DNA Reagent Kit (BD Biosciences, Bedford, MA, USA) according to the manufacturer's instructions and examined using the CyAn ADP Analyzer (Beckman Coulter, Brea, CA, USA). The percentages of cells in the G0/G1, S, and G2/M phases were calculated and compared. Experiments were performed in triplicate (Matsushita *et al*., 2015).

### 2.7 Apoptosis assays

Apoptotic cells were detected using the FITC Annexin V Apoptosis Detection Kit (BD Biosciences) according to the manufacturer's instructions and the BD FACSCelesta Flow Cytometer (BD Biosciences). Cells were identified as viable, dead, or early or late apoptotic cells, and the percentages of apoptotic cells under each experimental condition were compared. Anti‐poly (ADP‐ribose) polymerase (PARP) (#9542; Cell Signaling Technology, Danvers, MA, USA) was evaluated as a marker of apoptosis in this study (Idichi *et al*., 2018).

### 2.8 Western blotting

Western blotting was performed using a polyclonal anti‐PLOD1 antibody (1:1000 dilution; SAB1301577; Sigma‐Aldrich) and an anti‐glyceraldehyde 3‐phosphate dehydrogenase (GAPDH) antibody (1:10 000 dilution; ab8245; Abcam, Cambridge, UK) as a control (Fukumoto *et al*., 2014, 2015).

### 2.9 *miR‐140‐5p* and *miR‐140‐3p* localization within the RNA‐induced silencing complex (RISC) using Ago2 immunoprecipitation

T24 cells were transfected with 10 nm miRNA by reverse transfection. After 72 h, immunoprecipitation of the RISC was performed using the Ago2 miRNA isolation kit (Wako, Osaka, Japan). The expression levels of *miR‐140‐5p* and *miR‐140‐3p* in the immunoprecipitates were analyzed by qRT‐PCR. miRNA expression levels were normalized to that of *miR‐26a* (P/N: 000405; Applied Biosystems), which was not affected by *miR‐140‐5p* or *miR‐140‐3p* transfection.

### 2.10 Identification of candidate target genes regulated by miR‐140

To identify candidate target genes regulated by *miR‐140‐5p* and *miR‐140‐3p*, we used a combination of *in silico* and genome‐wide gene expression analyses. Genes potentially regulated by miRNAs in a sequence‐dependent manner are listed in the TargetScan database (release 7.2) (http://www.targetscan.org/vert_70/). Genes upregulated in BC were identified from a publicly available dataset in the Gene Expression Omnibus (GEO; accession number: GSE31684), and we narrowed down the list of candidate genes. Gene expression was also analyzed by our own oligonucleotide microarray analyses (Human GE 60K; Agilent Technologies), the data of which were deposited into the GEO (on June 14, 2018; http://www.ncbi.nlm.nih.gov/geo/) under accession number GSE115800.

### 2.11 Dual‐luciferase reporter assay

The wild‐type sequence of the *PLOD1* 3′‐untranslated region (UTR) was inserted between the *Sgf*I and *Pme*I restriction sites of the 3′‐UTR of the *hRluc* gene within the psiCHECK‐2 vector (C8021; Promega, Madison, WI, USA). We also generated *PLOD1* 3′‐UTR sequences containing deletions in the *miR‐140‐5p* target sites (positions 43–49 and 725–731) for insertion into the psiCHECK‐2 vector as described above. The psiCHECK‐2 vector was used as a cloning vector for the synthesized DNA sequences.

### 2.12 Immunohistochemistry

Immunohistochemistry procedures were performed according to a previously described method. Clinical tissue sections were incubated overnight at 4 °C with an anti‐PLOD1 antibody diluted 1:10 (SAB1301577; Sigma‐Aldrich).

### 2.13 Analysis of genes downstream of PLOD1

To investigate PLOD1‐regulated pathways in BC cells, we assessed gene expression changes in T24 and BOY cells transfected with the PLOD1 inhibitor. Microarray analysis was performed to obtain expression profiles in these cells, and the microarray data were deposited into the GEO (on December 4, 2018; accession number: GSE123318).

### 2.14 Analysis of the clinical significance of PLOD1 expression

We investigated the clinical importance of miRNAs and genes in BC patients using RNA‐sequencing data available in The Cancer Genome Atlas (TCGA; https://tcga-data.nci.nih.gov/tcga/). The gene expression and clinical data were obtained from cBioPortal (http://www.bioportal.org/), and provisional data were downloaded on October 5, 2018 (Anaya, 2016; Cerami *et al*., 2012; Gao *et al*., 2013).

### 2.15 Statistical analysis

Statistical comparisons involving two or three variables were performed using the Bonferroni‐adjusted Mann–Whitney *U*‐test. Spearman's rank tests were used to analyze the correlations among gene expression levels. These analyses were conducted using expert statview software (version 5.0, SAS Institute Inc., Cary, NC, USA). Multivariate analysis of prognostic factors for patient survival was conducted using jmp pro 13 (SAS Institute Inc.).

## 3 Results

### 3.1 Expression of *miR‐140‐5p* and *miR‐140‐3p* in BC tissues

**Figure 1:**
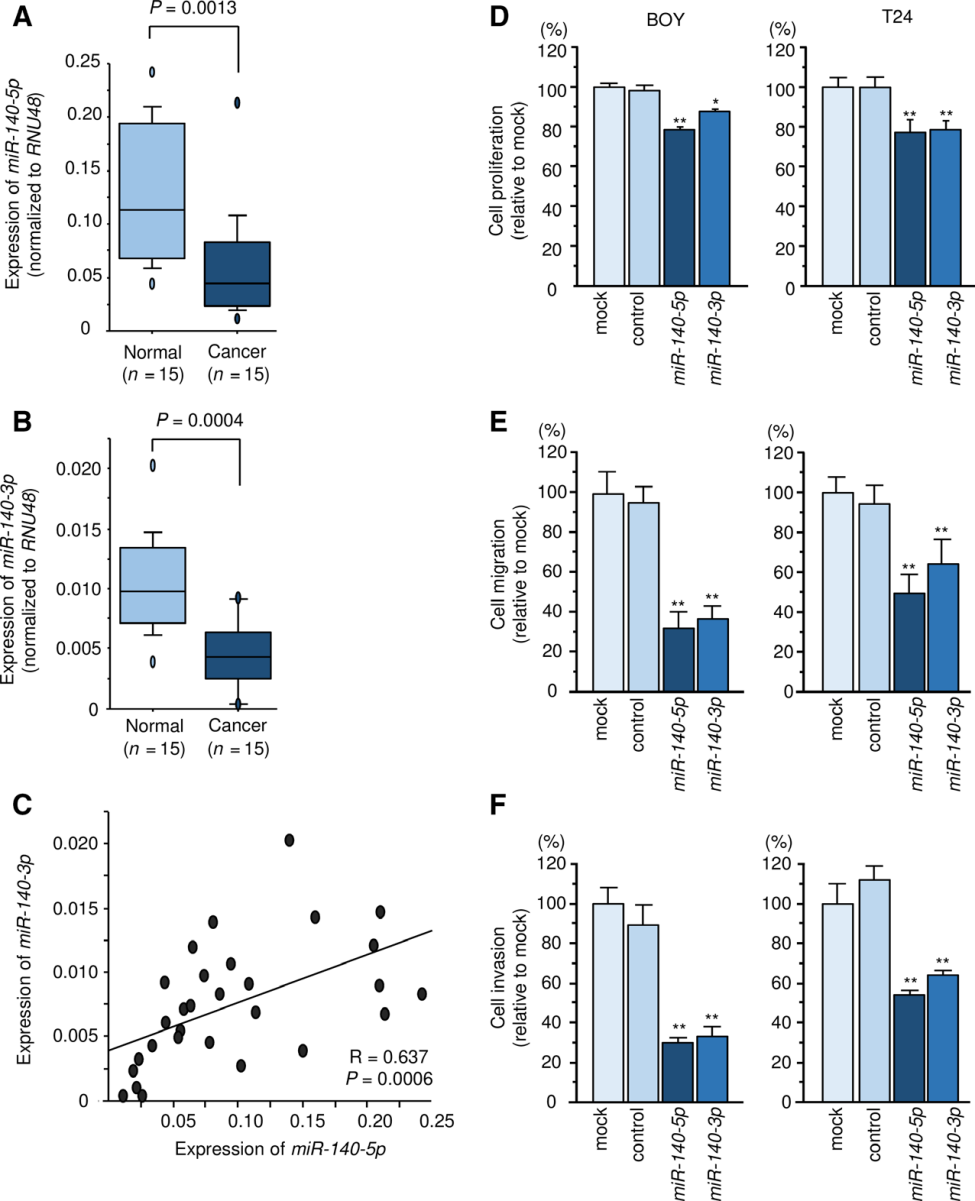
*miR‐140* expression and antitumor functions in BC. (A–C) Expression levels of *miR‐140‐5p* and *miR‐140‐3p* in BC clinical specimens (*P *=* *0.0013 and *P *=* *0.0004, respectively). *RNU48* was used as an internal control. *P*‐values were calculated using Bonferroni‐adjusted Mann–Whitney *U*‐test. A positive correlation between *miR‐140‐5p* and *miR‐140‐3p* expression levels was detected by Spearman's rank test (*R* = 0.637, *P *=* *0.0006). (D–F) Cell proliferation, migration, and invasion activities. Error bars are represented as mean ± SD (*n* = 5, *n* = 8, and *n* = 8, respectively). *P*‐values were calculated using Bonferroni‐adjusted Mann–Whitney *U*‐test. *P <* *0.001, **P <* *0.0001.

*hsa‐miR‐140* is located on chromosome 16q22.1 in humans. The mature sequences of *miR‐140‐5p* and *miR‐140‐3p* are 5′‐CAGUGGUUUUACCCUAUGGUAG‐3′ and 5′‐UACCACAGGGUAGAACCACGG‐3′, respectively. The expression levels of *miR‐140‐5p* and *miR‐140‐3p* were significantly downregulated in BC tissues compared with adjacent normal tissues (*P *=* *0.0013 and *P *=* *0.0004, respectively; Fig. 1A,B). Moreover, Spearman's rank test revealed a strong positive correlation between *miR‐140‐5p* and *miR‐140‐3p* expression levels (*R* = 0.637, *P *=* *0.0006; Fig. 1C).

### 3.2 Effect of *miR‐140‐5p* and *miR‐140‐3p* on the proliferation, migration, and invasion of BC cells

Restoration of *miR‐140‐5p* and *miR‐140‐3p* significantly suppressed BC cell proliferation, migration, and invasion abilities (Fig. 1D–F).

### 3.3 Effect of *miR‐140‐5p* and *miR‐140‐3p* on apoptosis and cell‐cycle assays in BOY cells

The percentage of apoptotic cells was significantly increased in *miR‐140‐5p‐* and *miR‐140‐3p*‐transfected cells compared with the control cells (Fig. S1A,B). Moreover, transfection of *miR‐140‐5p* and *miR‐140‐3p* upregulated the level of cleaved PARP (Fig. S1C). In a cell‐cycle analysis, the proportion of cells in the G0/G1 phase was significantly higher transfected with *miR‐140‐5p* compared with the control cells (Fig. S1D).

### 3.4 *miR‐140‐5p* and *miR‐140‐3p* localization within the RISC

We performed immunoprecipitation assays using antibodies targeting Ago2, which plays a pivotal role in the uptake of miRNAs into the RISC. After transfection of T24 cells with *miR‐140‐5p* and immunoprecipitation using anti‐Ago2 antibodies, *miR‐140‐5p* levels in the immunoprecipitates were significantly higher than those in the immunoprecipitates from mock‐ or miR‐control‐transfected cells as well as *miR‐140‐3p*‐transfected cells (*P *<* *0.0001; Fig. S2A). Similarly, after *miR‐140‐3p* transfection, substantial levels of *miR‐140‐3p* were detected in Ago2 immunoprecipitates compared with the controls (*P *<* *0.0001; Fig. S2B).

### 3.5 Candidate target genes of *miR‐140‐5p* and *miR‐140‐3p* in BC cells

**Figure 2:**
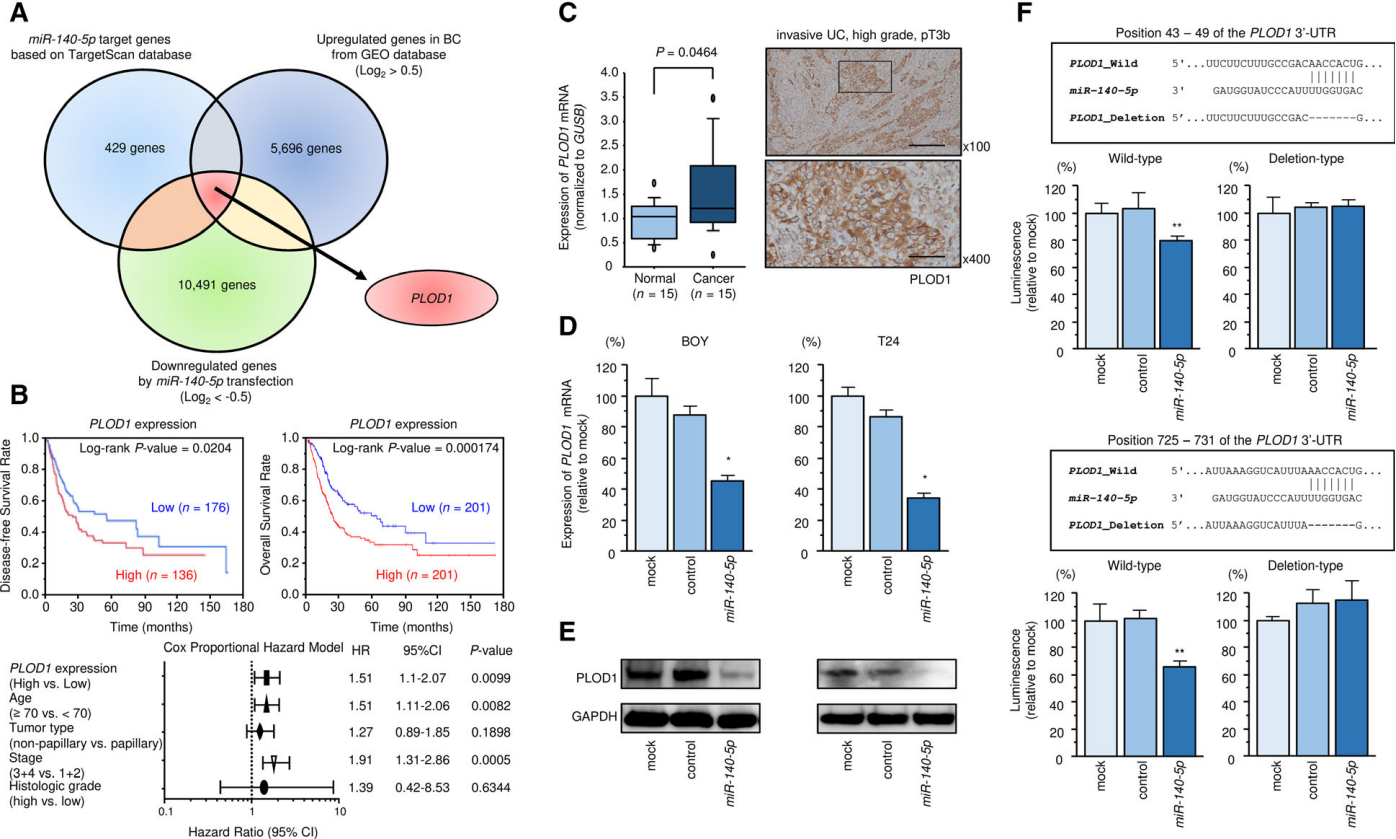
Clinical significance, expression, and regulation of PLOD1. (A) The strategy used to identify *miR‐140‐5p* candidate target genes, represented by a Venn diagram. (B) Clinical significance of PLOD1. (C) PLOD1 mRNA and protein expression in BC tissues. Scale bars of ×100 and ×400 represent 200 and 50 μm, respectively. *P*‐values were calculated using Bonferroni‐adjusted Mann–Whitney *U*‐test. (D) PLOD1 mRNA expression levels 48 h after transfection of BC cells with 10 nm *miR‐140‐5p*. *GAPDH* was used as the internal control gene. Error bars are represented as mean ± SD (*n* = 3). *P*‐values were calculated using Bonferroni‐adjusted Mann–Whitney *U*‐test. (E) PLOD1 protein expression 72 h after transfection with 10 nm *miR‐140‐5p*. GAPDH was used as the loading control. (F) Dual‐luciferase reporter assays using vectors encoding the wild‐type *PLOD1* 3′‐UTR sequence containing two putative *miR‐140‐5p* target sites and 3′‐UTR sequences with deletions of the target sites (Deletion). Normalized data were calculated as the ratio of *Renilla*/Firefly luciferase activities. Error bars are represented as mean ± SD (*n* = 3). *P*‐values were calculated using Bonferroni‐adjusted Mann–Whitney *U*‐test. *P <* *0.0001, **P <* *0.005.

We identified genes containing putative target sites for *miR‐140‐5p* and *miR‐140‐3p* within their 3′‐UTR sequence that also showed upregulated expression levels (log_2_ > 0.5) in BC tissues and downregulated expression levels (log_2_ < −0.5) in T24 cells transfected with *miR‐140‐5p* or *miR‐140‐3p* (Fig. 2A). Using this strategy, we identified 31 and 33 genes as candidate target genes of *miR‐140‐5p* and *miR‐140‐3p,* respectively (Table 1A and 1B). Among these genes, we focused on *PLOD1*, which was found to be a target of the *miR‐140‐5p* passenger strand.

**Table 1:** Candidate target genes of *miR‐140‐5p* and *miR‐140‐3p* in BC.

Gene symbol	Gene name	Entrez gene ID	Cytoband	GEO expression data fold change (tumor/normal)	*miR‐140‐5p* transfection in T24 (Log_2_ ratio)	Total binding sites	TCGA analysis for OS (high vs low expression: *P* value)
(A) *miR‐140‐5p*							
*CERCAM*	Cerebral endothelial cell adhesion molecule	51148	hs|9q34.11	1.928	−1.801	1	7.35E‐05
*PLOD1*	Procollagen‐lysine, 2‐oxoglutarate 5‐dioxygenase 1	5351	hs|1p36.22	2.150	−1.587	2	0.000174
*FADS1*	Fatty acid desaturase 1	3992	hs|11q12.2	1.741	−1.533	4	0.000384
*PAFAH1B2*	Platelet‐activating factor acetylhydrolase 1b, catalytic subunit 2 (30 kDa)	5049	hs|11q23.3	1.464	−0.595	1	0.0169
*PAX6*	Paired box 6	5080	hs|11p13	5.729	−0.550	1	0.0281
*TNN*	Tenascin N	63923	hs|1q25.1	2.521	−0.514	1	0.0622
*HDAC7*	Histone deacetylase 7	51564	hs|12q13.11	1.766	−0.750	1	0.0858
*BMP2K*	BMP2‐inducible kinase	55589	hs|4q21.21	2.025	−0.731	2	0.134
*PSRC1*	Proline/serine‐rich coiled‐coil 1	84722	hs|1p13.3	4.470	−0.655	1	0.157
*ZNF74*	Zinc finger protein 74	7625	hs|22q11.21	1.822	−0.508	1	0.211
*SOX4*	SRY (sex‐determining region Y)‐box 4	6659	hs|6p22.3	2.715	−0.816	1	0.256
*FRAS1*	Fraser extracellular matrix complex subunit 1	80144	hs|4q21.21	3.262	−1.122	1	0.297
*TSC22D2*	TSC22 domain family, member 2	9819	hs|3q25.1	1.520	−0.918	1	0.318
*GIT1*	G protein‐coupled receptor kinase interacting ArfGAP 1	28964	hs|17q11.2	3.992	−1.293	1	0.367
*YES1*	YES proto‐oncogene 1, Src family tyrosine kinase	7525	hs|18p11.32	1.734	−0.894	1	0.375
*MMD*	Monocyte to macrophage differentiation‐associated	23531	hs|17q22	2.736	−1.027	2	0.401
*SLC6A6*	Solute carrier family 6 (neurotransmitter transporter), member 6	6533	hs|3p25.1	1.781	−1.153	2	0.443
*FEN1*	Flap structure‐specific endonuclease 1	2237	hs|11q12.2	4.028	−0.941	1	0.446
*RALA*	v‐ral simian leukemia viral oncogene homolog A (ras related)	5898	hs|7p14.1	1.786	−2.318	1	0.462
*TTYH3*	Tweety family member 3	80727	hs|7p22.3	3.114	−1.493	2	0.61
*ZNF710*	Zinc finger protein 710	374655	hs|15q26.1	1.642	−0.501	1	0.649
*TTK*	TTK protein kinase	7272	hs|6q14.1	43.335	−0.520	2	0.686
*BCL2L1*	BCL2‐like 1	598	hs|20q11.21	2.118	−0.689	1	0.841
*PTP4A3*	Protein tyrosine phosphatase type IVA, member 3	11156	hs|8q24.3	2.455	−1.177	1	0.85
*RABIF*	RAB interacting factor	5877	hs|1q32.1	1.435	−0.871	1	0.889
*WASF1*	WAS protein family, member 1	8936	hs|6q21	2.068	−0.808	1	0.895
*ACSL6*	Acyl‐CoA synthetase long‐chain family member 6	23305	hs|5q31.1	1.995	−0.505	2	0.939
*LMNB1*	Lamin B1	4001	hs|5q23.2	10.537	−0.958	1	0.943
*C6orf47*	Chromosome 6 open reading frame 47	57827	hs|6p21.33	1.692	−1.018	1	0.0134^a^
*PROX2*	Prospero homeobox 2	283571	hs|14q24.3	5.328	−0.513	1	No data
*NT5C1A*	5′‐nucleotidase, cytosolic IA	84618	hs|1p34.2	5.445	−1.145	1	No data
(B) *miR‐140‐3p*							
*ADAM17*	ADAM metallopeptidase domain 17	6868	hs|2p25.1	2.062	−0.552	1	0.0033
*CCDC103*	Coiled‐coil domain containing 103	388389	hs|17q21.31	2.621	−2.588	2	0.0471
*PLXNA4*	Plexin A4	91584	hs|7q32.3	2.195	−0.639	1	0.0487
*THPO*	Thrombopoietin	7066	hs|3q27.1	3.383	−0.615	1	0.0611
*NR4A3*	Nuclear receptor subfamily 4, group A, member 3	8013	hs|9q22.33	5.420	−0.693	1	0.0904
*AEN*	Apoptosis‐enhancing nuclease	64782	hs|15q26.1	3.713	−0.602	1	0.101
*DBNL*	Drebrin‐like	28988	hs|7p13	2.543	−0.585	3	0.132
*GABRB2*	Gamma‐aminobutyric acid (GABA) A receptor, beta 2	2561	hs|5q34	1.944	−0.641	1	0.143
*FAM53B*	Family with sequence similarity 53, member B	9679	hs|10q26.13	1.880	−0.669	4	0.165
*COL7A1*	Collagen, type VII, alpha 1	1294	hs|3p21.31	2.370	−0.831	1	0.211
*SIRPA*	Signal‐regulatory protein alpha	140885	hs|20p13	1.573	−0.509	1	0.236
*ABCA12*	ATP‐binding cassette, subfamily A (ABC1), member 12	26154	hs|2q35	13.439	−0.505	1	0.271
*KCNK17*	Potassium channel, two‐pore domain subfamily K, member 17	89822	hs|6p21.2	1.633	−0.683	1	0.332
*KCTD16*	Potassium channel tetramerization domain containing 16	57528	hs|5q31.3	2.808	−0.702	2	0.431
*DAND5*	DAN domain family member 5, BMP antagonist	199699	hs|19p13.2	2.449	−0.529	1	0.481
*KIF5A*	Kinesin family member 5A	3798	hs|12q13.3	2.610	−0.691	4	0.592
*NUDT18*	Nudix (nucleoside diphosphate linked moiety X)‐type motif 18	79873	hs|8p21.3	2.690	−0.657	1	0.653
*SLC17A9*	Solute carrier family 17 (vesicular nucleotide transporter), member 9	63910	hs|20q13.33	1.976	−1.701	4	0.737
*HMGCS1*	3‐hydroxy‐3‐methylglutaryl‐CoA synthase 1 (soluble)	3157	hs|5p12	1.971	−1.187	2	0.808
*SNX22*	Sorting nexin 22	79856	hs|15q22.31	2.226	−0.676	2	0.861
*WDR55*	WD repeat domain 55	54853	hs|5q31.3	1.736	−0.646	1	0.942
*SRCIN1*	SRC kinase signaling inhibitor 1	80725	hs|17q12	3.306	−0.685	3	0.000248^a^
*BAI2*	Brain‐specific angiogenesis inhibitor 2	576	hs|1p35.2	1.864	−0.724	1	No data
*VGLL2*	Vestigial‐like family member 2	245806	hs|6q22.1	2.171	−0.670	1	No data
*NOL4*	Nucleolar protein 4	8715	hs|18q12.1	2.633	−1.014	1	No data
*MOBP*	Myelin‐associated oligodendrocyte basic protein	4336	hs|3p22.1	2.795	−0.676	1	No data
*CYLC1*	Cylicin, basic protein of sperm head cytoskeleton 1	1538	hs|Xq21.1	3.014	−0.616	1	No data
*ELAVL3*	ELAV‐like neuron‐specific RNA‐binding protein 3	1995	hs|19p13.2	3.171	−0.656	1	No data
*SCN1A*	Sodium channel, voltage‐gated, type I alpha subunit	6323	hs|2q24.3	3.609	−0.674	1	No data
*PAX7*	Paired box 7	5081	hs|1p36.13	3.899	−0.653	1	No data
*KCNK10*	Potassium channel, two‐pore domain subfamily K, member 10	54207	hs|14q31.3	3.933	−0.679	1	No data
*SVOP*	SV2‐related protein homolog (rat)	55530	hs|12q24.11	4.259	−0.670	1	No data
*CAMKV*	CaM kinase‐like vesicle‐associated	79012	hs|3p21.31	4.507	−1.615	4	No data

a Poor prognosis in patients with low expression.

### 3.6 Clinical significance and expression of PLOD1

Clinical data from BC patients were obtained from TCGA database, and information on survival revealed that patients with high *PLOD1* expression had a significantly poorer prognosis compared with patients with low expression (disease‐free survival: *P *=* *0.0204; overall survival: *P *=* *0.000174; Fig. 2B). High PLOD1 expression was also related to a highly malignant tumor morphology, advanced stage, and metastasis (Fig. S3A). According to multivariate Cox proportional hazards regression, high expression of PLOD1 was an independent predictive factor for overall survival in BC patients (hazard ratio: 1.51; 95% confidence interval: 1.1–2.07, *P *=* *0.0099) (Fig. 2B). PLOD1 mRNA expression levels were significantly upregulated in BC tissues compared with normal adjacent tissues (*P *=* *0.0464) (Fig. 2C). Immunostaining of PLOD1 in BC clinical specimens indicated high expression of PLOD1 in cancer lesions compared with adjacent noncancerous tissues at the same staining intensity (Fig. 2C).

In addition, expression levels of *PLOD2* and *PLOD3* were detected in BC clinical specimens (Fig. S4A,B). Also, immunohistochemical staining showed that overexpressed PLOD2 and PLOD3 were detected in cancer lesions (Fig. S4G,H). Interestingly, high expression of PLOD2 was significantly associated with poor prognosis of the patients with BC (Fig. S3B). Among *PLOD* family, expression of *PLOD1* was the highest in BC tissues (Fig. S4C). Clinicopathological analysis was performed between *PLODs* expression and BC (NMIBC or MIBC) clinical specimens. However, no significant association was found in this study (Fig. S4D–F).

### 3.7 PLOD1 was directly regulated by *miR‐140‐5p*

PLOD1 mRNA and protein levels were significantly decreased in T24 and BOY cells following transfection with *miR‐140‐5p* compared with mock‐transfected cells or those transfected with miR‐control (Fig. 2D,E). The TargetScan database indicated the presence of two *miR‐140‐5p* binding sites (positions 43–49 and 725–731) within the *PLOD1* 3′‐UTR. We performed luciferase reporter assays using a vector containing these sequences to assess whether *miR‐140‐5p* directly regulates *PLOD1* expression in a sequence‐dependent manner. Cotransfection of *miR‐140‐5p* with vectors harboring the *PLOD1* 3′‐UTR deletion constructs significantly decreased luciferase activity compared with the activity levels in mock‐transfected and miR‐control‐transfected cells (Fig. 2F).

### 3.8 Knockdown and rescue studies of PLOD1

**Figure 3:**
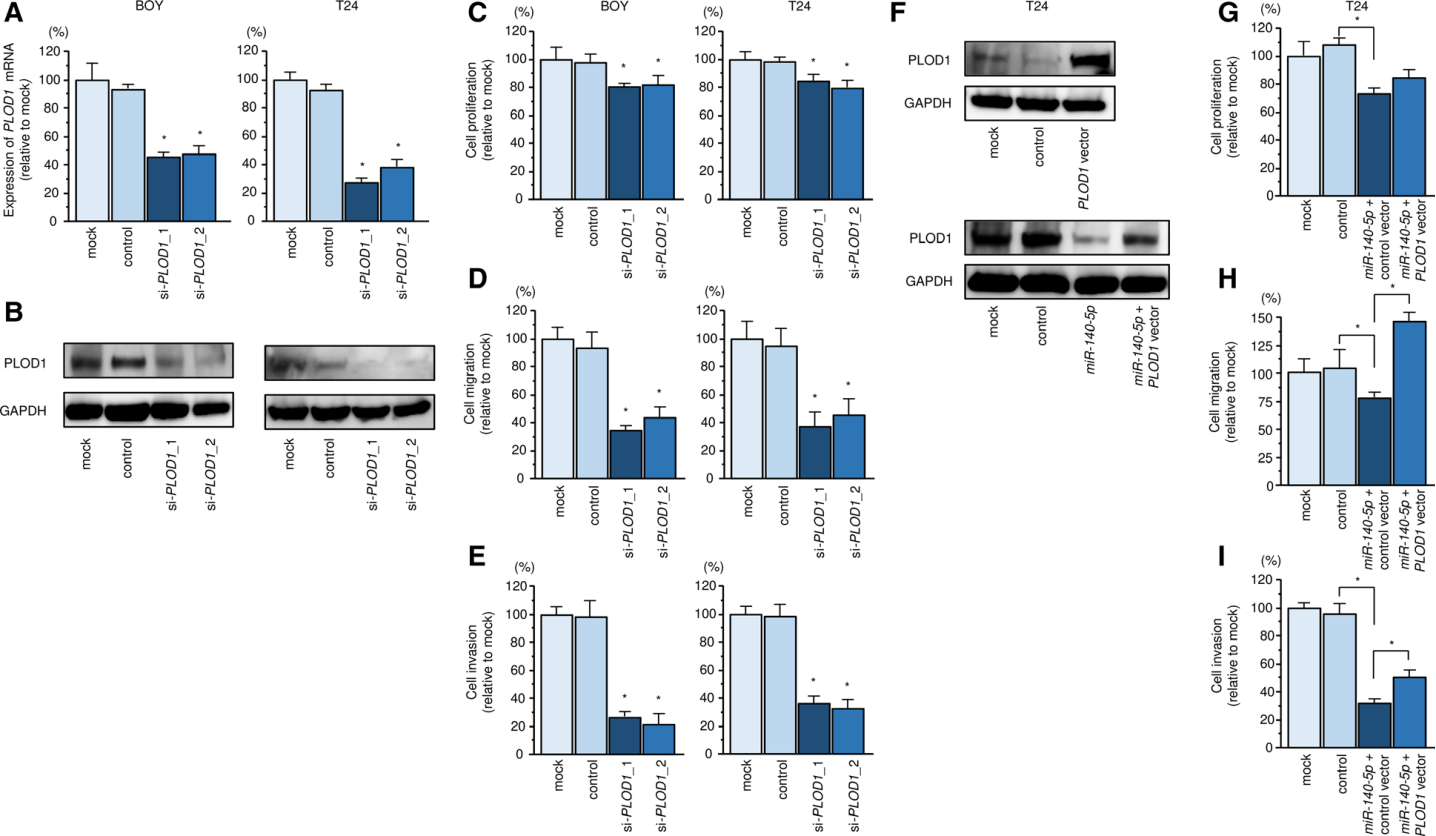
Knockdown and rescue studies of *PLOD1*. (A, B) PLOD1 mRNA and protein expression 72 h after transfection of si‐*PLOD1*_1 or si‐*PLOD1*_2 in BC cell lines. GAPDH was used as the control. Error bars are represented as mean ± SD (*n* = 3). *P*‐values were calculated using Bonferroni‐adjusted Mann–Whitney *U*‐test. (C) Cell proliferation, (D) migration, and (E) invasion activities in BC cells. Error bars are represented as mean ± SD (*n* = 5, *n* = 8, and *n* = 8, respectively). *P*‐values were calculated using Bonferroni‐adjusted Mann–Whitney *U*‐test. (F) PLOD1 protein expression was evaluated 72 h after reverse transfection of *miR‐140‐5p* and 48 h after forward transfection of *PLOD1*. GAPDH was used as the loading control. (G) Cell proliferation assay performed 72 h after reverse transfection of *miR‐140‐5p* and 48 h after forward transfection of *PLOD1*. (H) Cell migration assay performed 48 h after reverse transfection of *miR‐140‐5p* and 24 h after forward transfection of *PLOD1*. (I) Cell invasion assay performed 48 h after reverse transfection of *miR‐140‐5p* and 24 h after forward transfection of *PLOD1*. Error bars are represented as mean ± SD (*n* = 5, *n* = 8, and *n* = 8, respectively). *P*‐values were calculated using Bonferroni‐adjusted Mann–Whitney *U*‐test. *P <* *0.0001.

We confirmed that both PLOD1 mRNA and protein expression levels were suppressed by siRNA‐mediated PLOD1 knockdown in BC cells (Fig. 3A and B). Transfection of si‐*PLOD1* suppressed cell proliferation, migration, and invasion activities (Fig 3C–E). The percentage of apoptotic cells was significantly increased in si‐*PLOD1‐*transfected cells compared with the control cells (Fig. S5A,B). Moreover, transfection of si‐*PLOD1* upregulated the level of cleaved PARP (Fig. S5C). In a cell‐cycle analysis, the proportion of cells in the G0/G1 phase was significantly higher, transfected with si‐*PLOD1*_2 compared with the control cells, although G2/M phase was significantly elevated in si‐*PLOD1*_1 transfection (Fig. S5D).

In addition, we performed a PLOD1 rescue study in T24 cells to validate whether oncogenic pathways regulated by PLOD1/*miR‐140‐5p* are crucial for BC development. *PLOD1* and *miR‐140‐5p* transfection restored PLOD1 protein expression (Fig. 3F). Functional assays demonstrated that BC cell migration and invasion were significantly recovered by *PLOD1* and *miR‐140‐5p* transfection compared with *miR‐140‐5p* alone (Fig. 3G–I).

### 3.9 Functional analysis of a PLOD1 inhibitor

**Figure 4:**
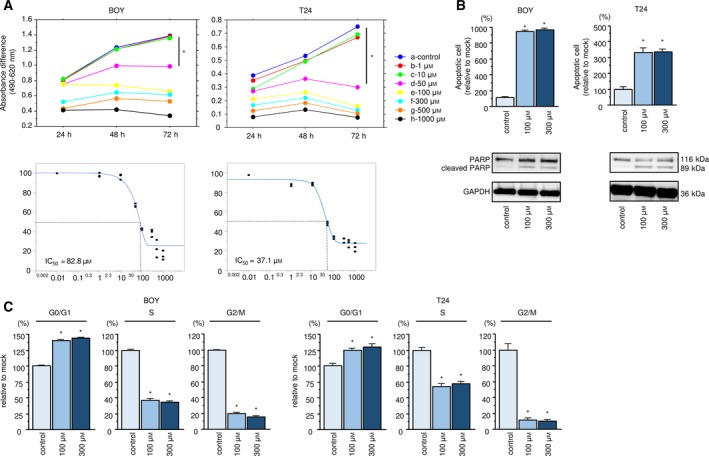
Functional analysis of a PLOD1 inhibitor. (A) Cell proliferation assay of BC cells transfected with an inhibitor of PLOD1 and the IC_50_ values of the PLOD1 inhibitor. IC_50_ values were calculated using jmp software. Error bars are represented as mean ± SD (*n* = 5). *P*‐values were calculated using Bonferroni‐adjusted Mann–Whitney *U*‐test. (B) Effect of the PLOD1 inhibitor on apoptosis, as assessed by apoptosis assays, and western blot analysis of cleaved PARP, as a marker of apoptosis. GAPDH was used as the loading control. Error bars are represented as mean ± SD (*n* = 3). *P*‐values were calculated using Bonferroni‐adjusted Mann–Whitney *U*‐test. (C) Effect of the PLOD1 inhibitor on the cell cycle. The bar charts represent the percentages of inhibitor‐transfected cells relative to the control cells in the G0/G1, S, and G2/M phases, respectively. Error bars are represented as mean ± SD (*n* = 3). *P*‐values were calculated using Bonferroni‐adjusted Mann–Whitney *U*‐test. *P <* *0.0001.

After transfection of the PLOD1 inhibitor 2,2′‐dipyridyl into BC cells, cell proliferation was suppressed in a dose‐dependent manner (Fig. 4A). The IC_50_ of 2,2′‐dipyridyl was 82.8 μm in BOY cells and 37.1 μm in T24 cells. Cell migration and invasion were also decreased in a dose‐dependent manner in cells transfected with the inhibitor (Fig. S6). In addition, the percentage of apoptotic cells was increased in PLOD1 inhibitor‐transfected cells compared with the control cells (Fig. 4B). Moreover, transfection of PLOD1 inhibitor upregulated the level of cleaved PARP (Fig. 4B). In a cell‐cycle analysis, the proportion of cells in the G0/G1 phase was significantly higher in BC cells transfected with the PLOD1 inhibitor compared with the control cells (Fig. 4C). In addition, we confirmed that the inhibitor suppressed the mRNA and protein levels of PLOD1 in a dose‐dependent manner (Fig. S7). Apoptosis and cell‐cycle experiments gave similar results in BOY cells (Fig. S8).

### 3.10 Genes affected by the PLOD1 inhibitor

PLOD1 acts as lysyl hydroxylases that catalyze hydroxylation of collagen lysines, and it works under the following conditions, extracellular matrix maturation and remodeling. In order to explore the functional significance of *PLOD1* on tumor progression, we examined the *PLOD1*‐mediated downstream genes and pathways. As shown in the Venn diagram in Fig. S9, 1518 genes were considerably downregulated after transfection of the PLOD1 inhibitor in BOY and T24 cells. In a KEGG analysis of these genes, we identified 39 pathways enriched among the PLOD1‐affected genes, including pathways related to cell cycle and apoptosis (Table 2).

**Table 2:** Molecular pathways significantly enriched among the genes affected by PLOD1 inhibitor treatment in BC cells.

Number of genes	Annotations	*P*‐value
18	(KEGG) 04110: Cell cycle	2.37E‐05
28	(KEGG) 05200: Pathways in cancer	1.90E‐04
13	(KEGG) 04512: ECM–receptor interaction	2.36E‐04
15	(KEGG) 04114: Oocyte meiosis	3.29E‐04
7	(KEGG) 00760: Nicotinate and nicotinamide metabolism	4.73E‐04
13	(KEGG) 05146: Amebiasis	1.02E‐03
8	(KEGG) 00310: Lysine degradation	4.59E‐03
6	(KEGG) 00410: Beta‐alanine metabolism	4.74E‐03
15	(KEGG) 00230: Purine metabolism	5.32E‐03
6	(KEGG) 00640: Propanoate metabolism	1.00E‐02
10	(KEGG) 04914: Progesterone‐mediated oocyte maturation	1.06E‐02
10	(KEGG) 04540: Gap junction	1.06E‐02
6	(KEGG) 03030: DNA replication	1.21E‐02
9	(KEGG) 04070: Phosphatidylinositol signaling system	1.24E‐02
16	(KEGG) 04510: Focal adhesion	1.32E‐02
10	(KEGG) 04916: Melanosis	1.84E‐02
9	(KEGG) 05222: Small‐cell lung cancer	2.10E‐02
14	(KEGG) 04020: Calcium signaling pathway	2.26E‐02
11	(KEGG) 04142: Lysosome	2.34E‐02
4	(KEGG) 00670: One carbon pool by folate	2.51E‐02
9	(KEGG) 05414: Dilated cardiomyopathy	2.54E‐02
4	(KEGG) 00100: Steroid biosynthesis	2.58E‐02
6	(KEGG) 00280: Valine, leucine, and isoleucine degradation	2.68E‐02
6	(KEGG) 04962: Vasopressin‐regulated water reabsorption	2.68E‐02
14	(KEGG) 04062: Chemokine signaling pathway	2.73E‐02
8	(KEGG) 04146: Peroxisome	2.83E‐02
6	(KEGG) 00561: Glycerolipid metabolism	2.97E‐02
5	(KEGG) 03410: Base excision repair	3.06E‐02
11	(KEGG) 04910: Insulin signaling pathway	3.08E‐02
8	(KEGG) 04974: Protein digestion and absorption	3.08E‐02
6	(KEGG) 04961: Endocrine and other factor‐regulated calcium reabsorption	3.43E‐02
8	(KEGG) 04350: TGF‐beta signaling pathway	3.52E‐02
4	(KEGG) 03430: Mismatch repair	3.87E‐02
8	(KEGG) 04210: Apoptosis	4.40E‐02
6	(KEGG) 00590: Arachidonic acid metabolism	4.46E‐02
10	(KEGG) 04724: Glutamatergic synapse	4.46E‐02
4	(KEGG) 00563: Glycosylphosphatidylinositol(GPI)‐anchor biosynthesis	4.68E‐02
6	(KEGG) 05217: Basal cell carcinoma	4.91E‐02
4	(KEGG) 03440: Homologous recombination	4.98E‐02

BC, bladder cancer.

## 4 Discussion

RNA sequencing is a suitable technology for creating miRNA expression signatures in cancer cells. Analyses of our miRNA signatures in cancers revealed that the passenger strand of some miRNA duplexes is functional in cancer cells by targeting cancer‐related genes (Arai *et al*., 2018b; Goto *et al*., 2017; Matsushita *et al*., 2015; Sugawara *et al*., 2018; Yamada *et al*., 2018a,2018b). This makes it possible to identify novel cancer pathways based on aberrantly expressed passenger strand miRNAs.

In this study, we focused on both strands of pre‐*miR‐140* (*miR‐140‐5p* and *miR‐140‐3p*) and revealed their antitumor functions in BC cells. Previous reports showed that *miR‐140‐3p* is downregulated in squamous cell lung cancer and functions as a tumor suppressor by targeting bromodomain containing 9 *in vitro* and *in vivo* (Huang *et al*., 2019). As with *miR‐140‐3p*, a tumor‐suppressive function of *miR‐140‐5p* has been reported in several cancers. *miR‐140‐5p* exerted a tumor‐suppressive function and enhanced the effect of existing therapeutic drugs in non‐small‐cell lung cancer (Flamini *et al*., 2017). Another report showed that *miR‐140‐5p* suppressed cell aggressiveness and suggested that *miR‐140‐5p* is a prognostic marker in gastric cancer (Fang *et al*., 2017). Downregulation of miRNAs was reported to be caused by epigenetic factors such as DNA methylation or histone deacetylation. Previous study showed that suppression of *miR‐140* expression was influenced by the hypermethylation of the promoter region in breast cancer (Wolfson *et al*., 2014). Elucidation of the detailed molecular mechanism of downregulation of *miR‐140‐5p* and *miR‐140‐3p* is also essential in BC cells. These studies indicate that both strands of pre‐*miR‐140* act as critical miRNAs that prevent malignant transformation in cells. To our knowledge, this is the first study to identify a functional role of the *miR‐140* duplex and its oncogene targets in BC.

Our next focus was to investigate the molecular networks regulated by these miRNAs in BC cells. A total of 31 genes regulated by *miR‐140‐5p* and 33 genes regulated by *miR‐140‐3p* were identified as putative oncogenic targets in BC cells. Among these targets, the expression levels of eight genes (*CERCAM*,* PLOD1*,* FADS1*,* PAFAH1B2*,* PAX6*,* ADAM17*,* CCDC103,* and *PLXNA4*) were closely associated with BC pathogenesis. These genes are promising as therapeutic targets and prognostic markers, and further analysis is necessary to elucidate the molecular pathogenesis of BC. We focused on *PLOD1* to investigate its oncogenic functions and clinical significance in BC. *PLOD* genes encode lysyl hydroxylases, which are crucial for collagen biosynthesis, cross‐linking, and deposition (Qi and Xu, 2018). Collagen is a major component of the extracellular matrix (ECM), and collagen cross‐linking is related to the stiffness of the ECM, which enhances cancer cell migration, invasion, and focal adhesion (Du *et al*., 2017; Peinado *et al*., 2008). The PLOD family consists of PLOD1, PLOD2, and PLOD3. A number of studies have demonstrated that overexpression of PLOD2 and PLOD3 promotes cancer progression and metastasis. Our previous studies showed that aberrant expression of *PLOD2* was detected in BC and renal cell carcinoma tissues, and its overexpression enhanced cancer cell malignant transformation (Kurozumi *et al*., 2016; Miyamoto *et al*., 2016). We hypothesized that members of the *PLOD* family member are deeply involved in the molecular pathogenesis of BC. On the other hand, there are not many reports on the role of PLOD1 in cancer (Qi and Xu, 2018). Previous studies showed that aberrant expression of PLOD1 was significantly associated with shorter survival in patients with gastric or colorectal cancer (Wang *et al*., 2018). Overexpression of PLOD1 was also detected in esophageal squamous cell carcinoma and breast cancer (Gilkes *et al*., 2013; Li *et al*., 2017). Mutations in *PLOD1* are the cause of PLOD1‐related kyphoscoliotic Ehlers–Danlos syndrome, an autosomal recessive generalized connective tissue disorder (Giunta *et al*., 2005).

The data from a large number of cohort analyses in TCGA database show that high expression of *PLOD1* is significantly associated with a poor prognosis (overall survival: *P *=* *0.000174), more strongly than are *PLOD2* and *PLOD3* (OS: *P *=* *0.0097 and *P *=* *0.315, respectively) (Fig. S3B,C). Furthermore, multivariate analysis showed that PLOD1 expression was an independent prognostic factor in patients with BC (hazard ratio = 1.51, *P *=* *0.0099). Moreover, high expression of *PLOD1* was significantly associated with tumor stage and presence of metastasis. Aberrant expression of *PLOD1* has been shown to be closely related to the malignant phenotype of BC. Development of a new diagnostic strategy for BC using PLOD1 expression as a marker is desired.

Aberrant expression of PLOD1 was detected in BC clinical specimens, and inhibition of PLOD1 by siRNA‐mediated knockdown or treatment with a PLOD1 inhibitor significantly reduced the malignant phenotype of BC cells (e.g., decreases in proliferation, migration, and invasion and an increase in apoptosis). We used 2,2′‐dipyridyl, an iron chelator, as an inhibitor of PLOD1 in this study (Bernardes *et al*., 2018; Jover *et al*., 2018). Collagen lysyl hydroxylases reportedly depend on Fe2+ binding for stabilization, and 2,2′‐dipyridyl prevents prolyl and lysyl hydroxylation (Barsh and Byers, 1981; Guo *et al*., 2018). A previous report showed that inhibition of PLOD1 and lysyl oxidase suppressed arterial smooth muscle cell calcification via ECM remodeling (Jover *et al*., 2018). Another study was conducted to investigate the effect of 2,2′‐dipyridyl in combination with doxorubicin in breast cancer cells (Bernardes *et al*., 2018). In this study, we showed that PLOD1 expression and cell proliferation were suppressed after transfection of a PLOD1 inhibitor in a dose‐dependent manner. Moreover, the PLOD1 inhibitor induced apoptosis and cell‐cycle arrest at the G1‐to‐S phase transition.

The molecular mechanism of the antitumor effect of the PLOD1 inhibitor in BC cells was evaluated by global gene expression analysis. As a result, genes associated with cell cycle, ECM–receptor interactions, and apoptosis were differentially expressed in cells transfected with the PLOD1 inhibitor, supporting our current data. We focused on several genes (e.g., *CCNB1*,* CCNB2,* and *SKP2*) involved in ‘cell‐cycle pathway’. Expression of *CCNB2* (cyclin B2) was upregulated in BC tissues, and suppression of its expression significantly inhibited invasive and metastatic abilities (Lei *et al*., 2016). Our recent study showed that *CCNB1* (cyclin B1) was regulated by antitumor *miR‐223‐5p* in BC cells and its high expression was closely associated with poor prognosis of the patients with BC by TCGA database analysis (Sugawara *et al*., 2018). Moreover, overexpression of *SKP2* (S‐phase kinase‐associated protein 2) was significantly related to advanced tumor stage and grade of the patients with BC (Kawakami *et al*., 2007).

Moreover, we performed rescue experiments by overexpressing *PLOD1* and *miR‐140‐5p*. The results revealed that PLOD1 can counteract the antitumor effects, in terms of cell migration and invasion, of *miR‐140‐5p* in BC cells, indicating that the PLOD1/*miR‐140‐5p* axis plays an important role in BC development.

## 5 Conclusion

Both strands of the *miR‐140* duplex (*miR‐140‐5p* and *miR‐140‐3p*) suppressed BC cell malignant transformation. Genes controlled by the *miR‐140‐5p* were found to be related to BC pathogenesis. PLOD1 expression was directly regulated by the *miR‐140‐5p* in BC cells. Aberrant expression of PLOD1 was closely contributed to BC development. Furthermore, inhibition of PLOD1 expression significantly attenuated to BC cell aggressive phenotypes. PLOD1 might be a novel biomarker and therapeutic target in BC. Further investigation is required for clinical application.

## Conflict of interest

The authors declare no conflict of interest.

## Author contributions

YY, NS, and TI designed the whole study and wrote the manuscript. MK, TA, HS, AU, SM, SS, and AK contributed to experimental design and data collection. All authors have agreed with the manuscript and provide their consent for publication.

## Supporting information


**Fig. S1.** Effect of *miR‐140‐5p* and *miR‐140‐3p* on apoptosis and cell‐cycle assays in BOY cells.

**Fig. S2.** *miR‐140‐5p* and *miR‐140‐3p* localization within the RISC.

**Fig. S3.** Clinical database analysis of *PLOD1*,* PLOD2* and *PLOD3* expression in BC patients.

**Fig. S4.** Expression analysis of PLOD1, PLOD2 and PLOD3 in BC tissues.

**Fig. S5.** Effect of *si*‐PLOD1 on apoptosis and cell‐cycle assays in BOY cells.

**Fig. S6.** Effect of a PLOD1 inhibitor on the migration and invasion of BC cells.

**Fig. S7.** Effect of a PLOD1 inhibitor on PLOD1 expression.

**Fig. S8.** Effect of a PLOD1 inhibitor on apoptosis and cell‐cycle assays in BC cells.

**Fig. S9.** Downstream pathways affected by treatment with a PLOD1 inhibitor in BC cells.

**Table S1.** Background characteristics of the BC patients.

## Data Availability

Data acquired during the course of this study are available in GEO: GSE115800 and GSE123318.
