# Supplementary figures and images for: Aberrantly expressed PLOD1 promotes cancer aggressiveness in bladder cancer: a potential prognostic marker and therapeutic target
Source: Mol Oncol. 2019 Jun 27;13(9):1898–912. doi: 10.1002/1878-0261.12532 (PMC6717764; doi:10.1002/1878-0261.12532)

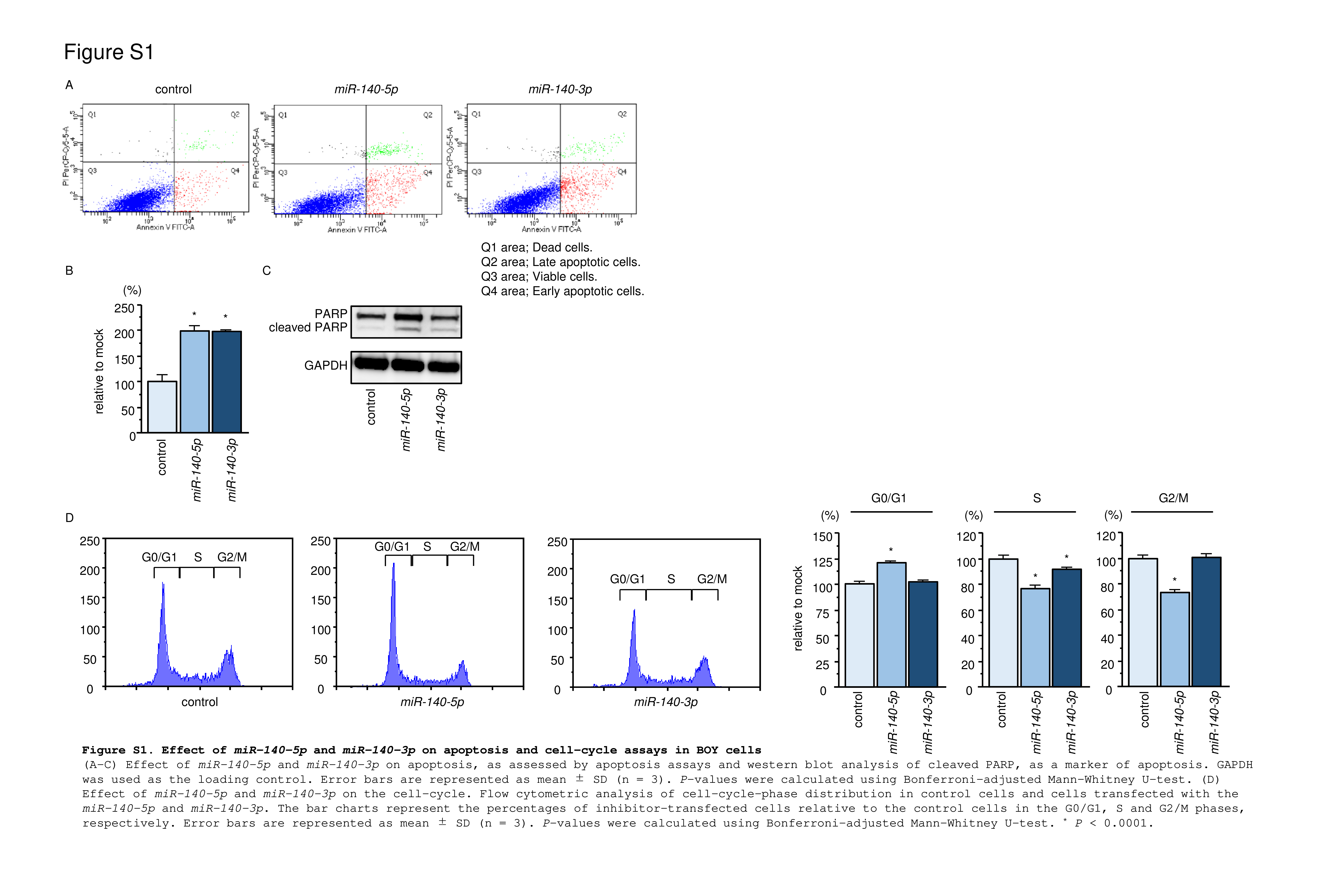

Supplement: Supplementary file 1 — Fig. S1. Effect of miR‐140‐5p and miR‐140‐3p on apoptosis and cell‐cycle assays in BOY cells. [file MOL2-13-1898-s001.tiff]

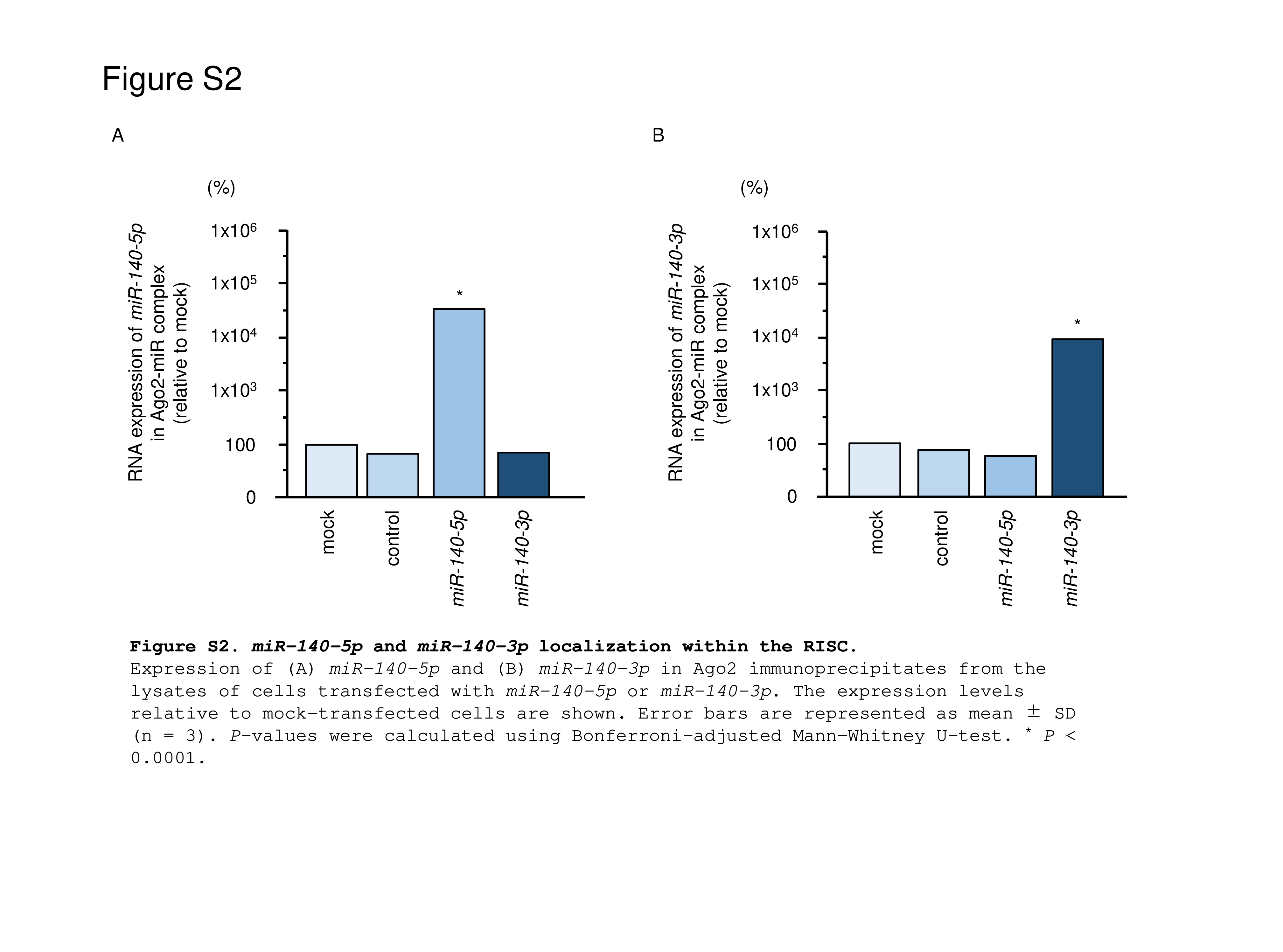

Supplement: Supplementary file 2 — Fig. S2. miR‐140‐5p and miR‐140‐3p localization within the RISC. [file MOL2-13-1898-s002.tiff]

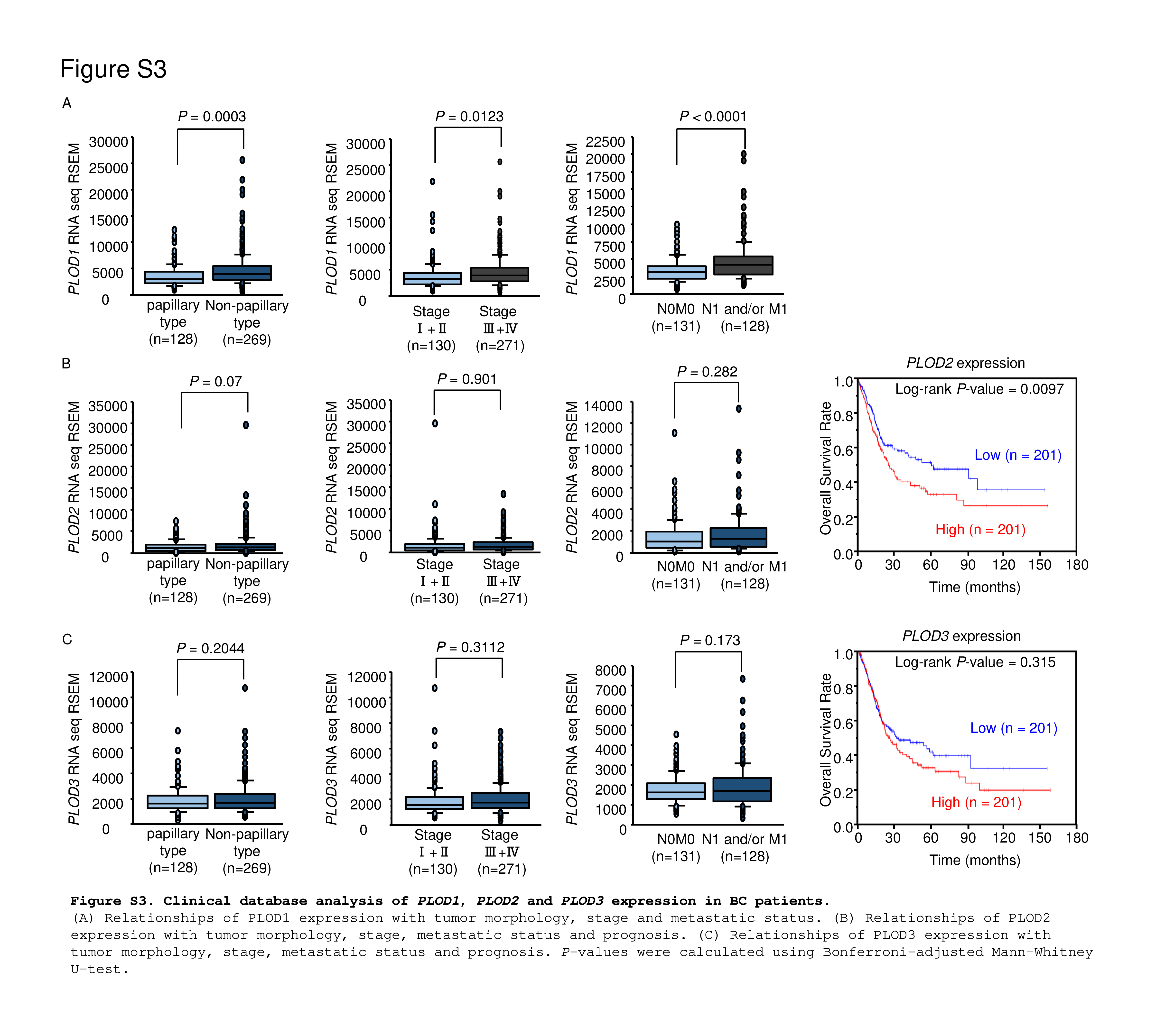

Supplement: Supplementary file 3 — Fig. S3. Clinical database analysis of PLOD1, PLOD2 and PLOD3 expression in BC patients. [file MOL2-13-1898-s003.tiff]

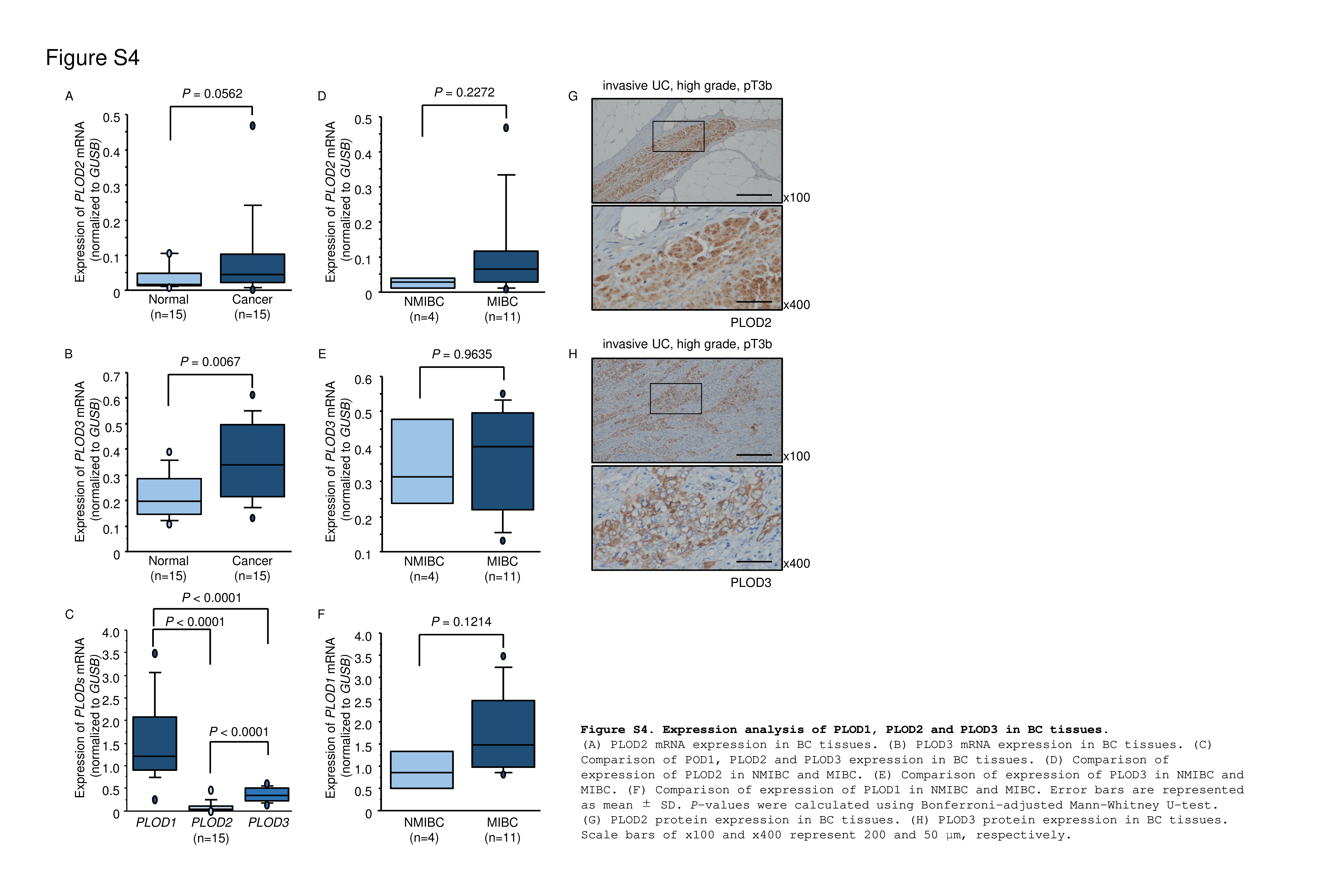

Supplement: Supplementary file 4 — Fig. S4. Expression analysis of PLOD1, PLOD2 and PLOD3 in BC tissues. [file MOL2-13-1898-s004.tiff]

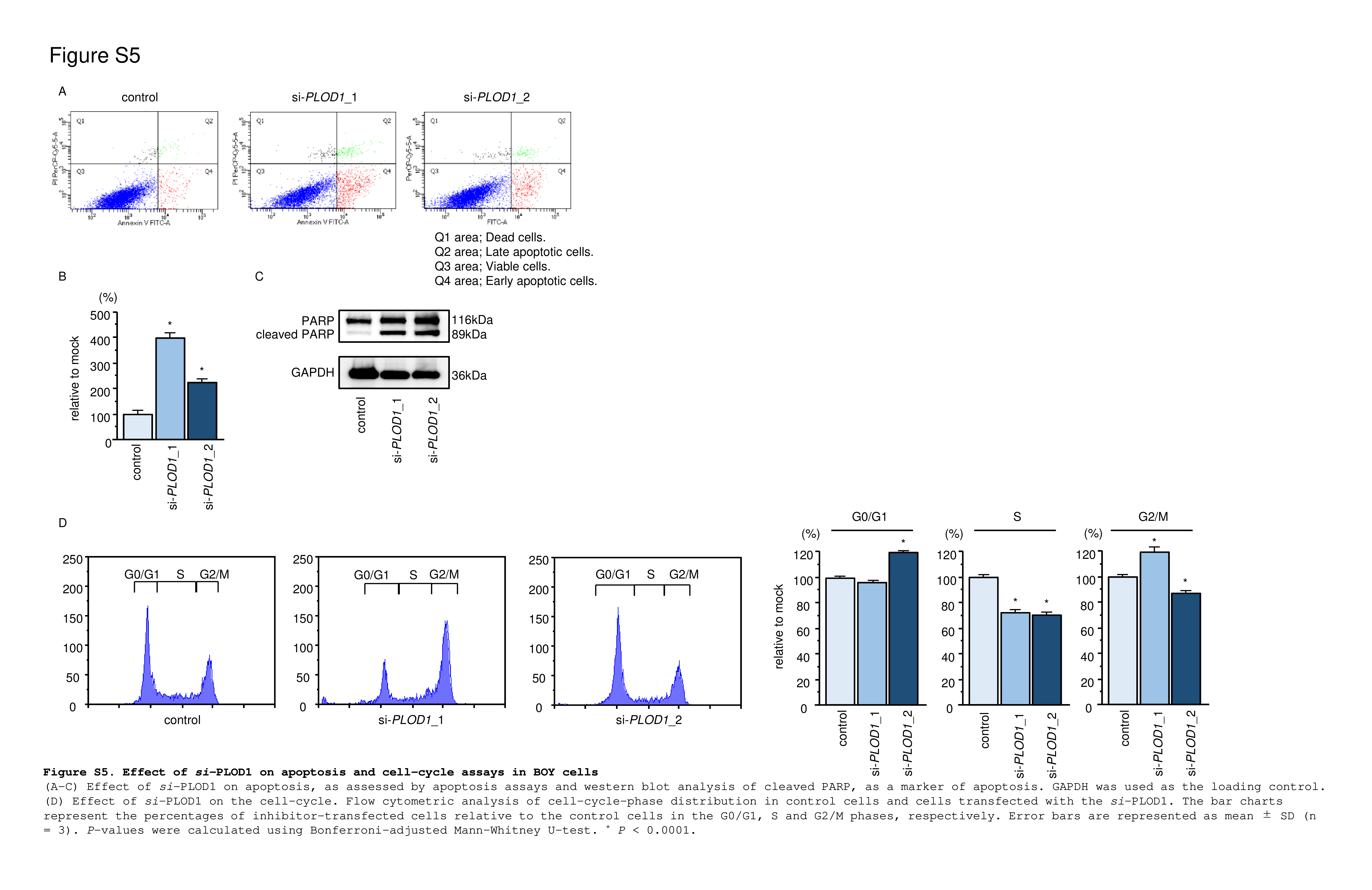

Supplement: Supplementary file 5 — Fig. S5. Effect of si‐PLOD1 on apoptosis and cell‐cycle assays in BOY cells. [file MOL2-13-1898-s005.tiff]

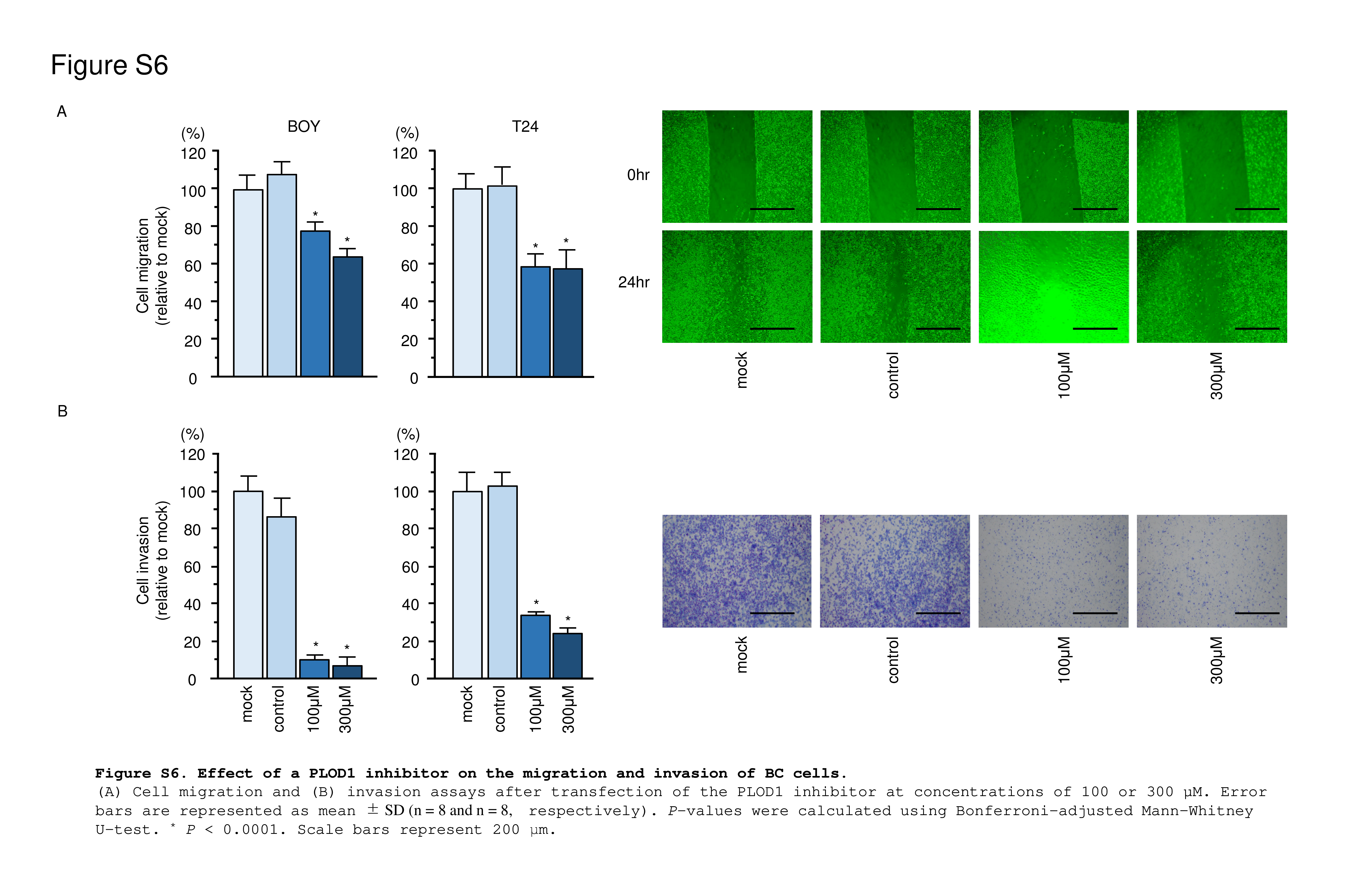

Supplement: Supplementary file 6 — Fig. S6. Effect of a PLOD1 inhibitor on the migration and invasion of BC cells. [file MOL2-13-1898-s006.tiff]

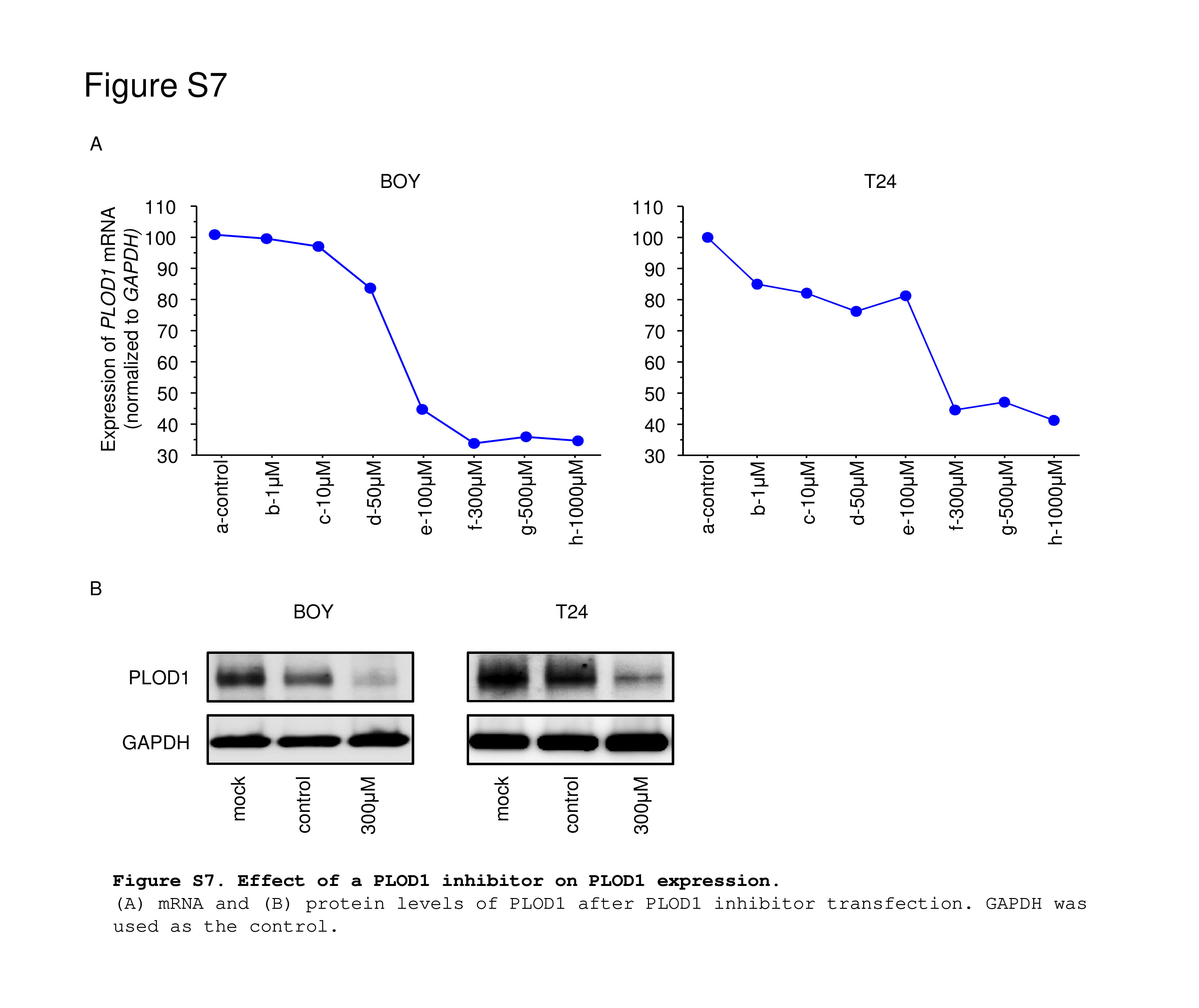

Supplement: Supplementary file 7 — Fig. S7. Effect of a PLOD1 inhibitor on PLOD1 expression. [file MOL2-13-1898-s007.tiff]

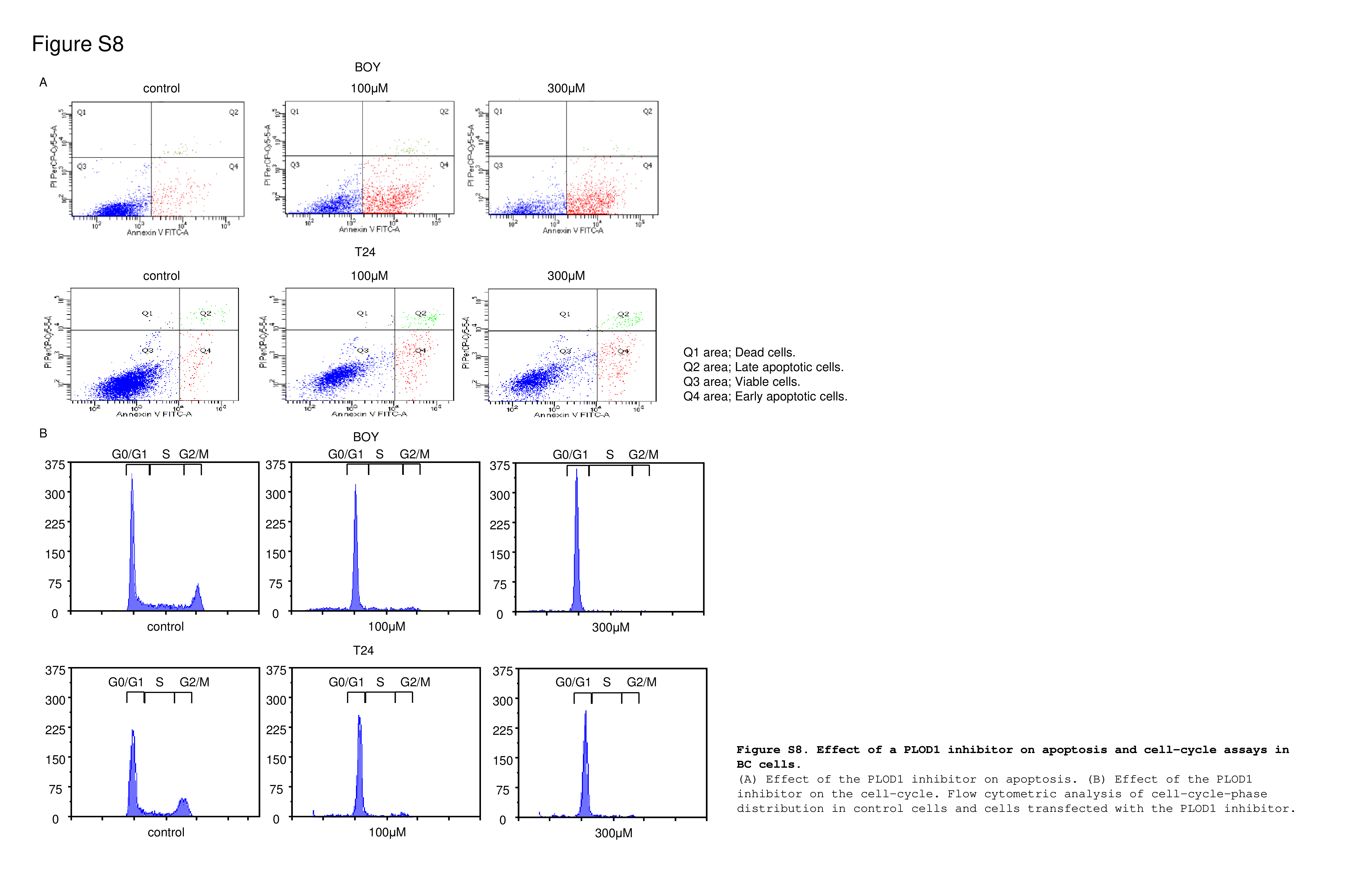

Supplement: Supplementary file 8 — Fig. S8. Effect of a PLOD1 inhibitor on apoptosis and cell‐cycle assays in BC cells. [file MOL2-13-1898-s008.tiff]

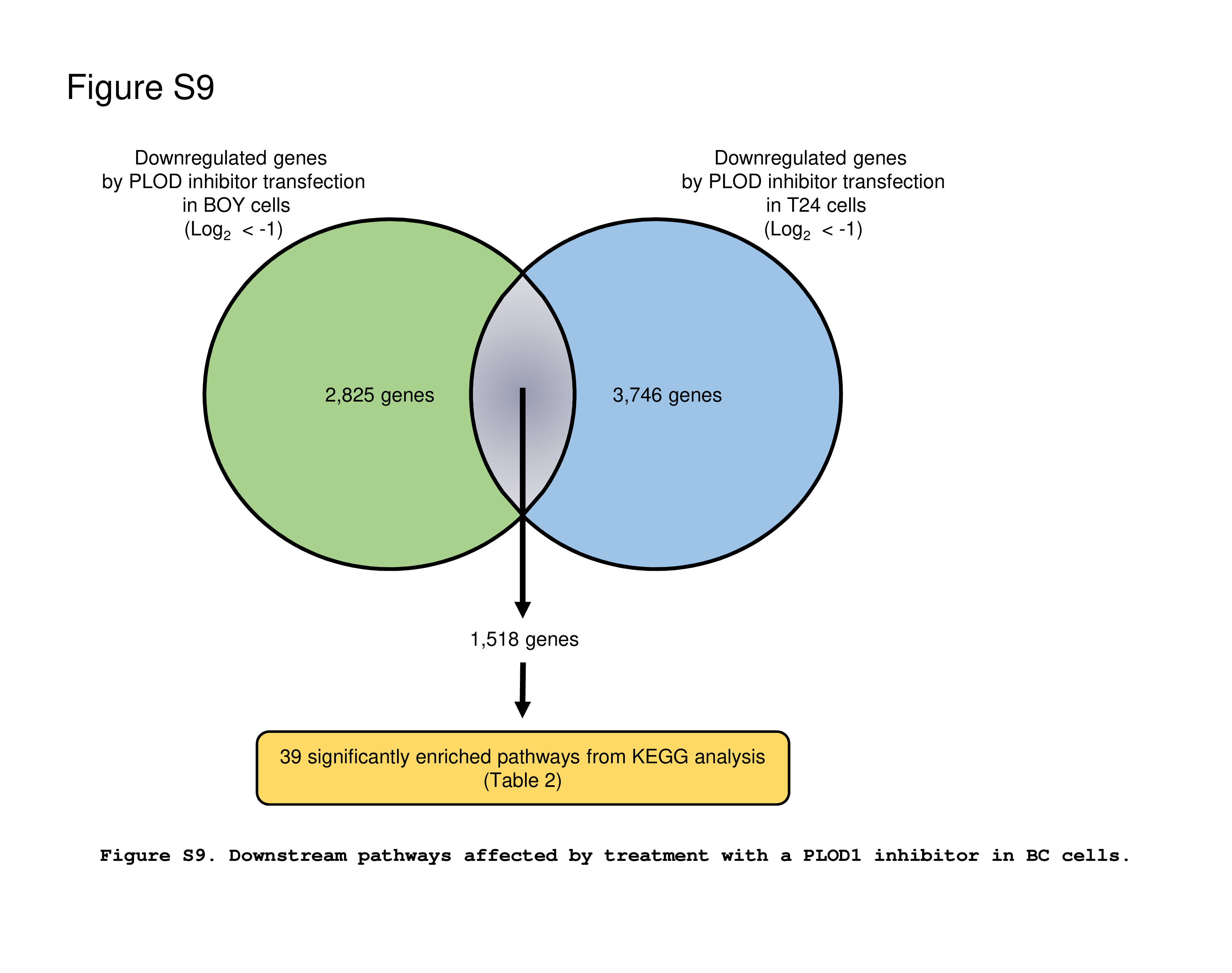

Supplement: Supplementary file 9 — Fig. S9. Downstream pathways affected by treatment with a PLOD1 inhibitor in BC cells. [file MOL2-13-1898-s009.tiff]
