# Supplementary material for: Aberrantly expressed PLOD1 promotes cancer aggressiveness in bladder cancer: a potential prognostic marker and therapeutic target
Source: Mol Oncol. 2019 Jun 27;13(9):1898–912. doi: 10.1002/1878-0261.12532 (PMC6717764; doi:10.1002/1878-0261.12532)
Supplement: Supplementary file 10 — Table S1. Background characteristics of the BC patients. [file MOL2-13-1898-s010.docx]

Table S1. Background characteristics of the BC patients.

| Number | 15 |
| --- | --- |
| Median Age (range)  Gender | 73 (51-81) |
| male | 12 (80%) |
| female  Pathological type | 3 (20%) |
| non-invasive UC | 1 (6.7%) |
| invasive UC | 13 (86.7%) |
| CIS  Grade | 1 (6.7%) |
| 2 | 1 (6.7%) |
| 3  pT stage | 14 (93.3%) |
| Tis | 1 (6.7%) |
| Ta | 1 (6.7%) |
| T1 | 2 (13.3%) |
| T2 | 4 (26.7%) |
| T3 | 7 (46.7%) |

BC; bladder cancer
